# Nationwide expansion of the EGUIDE project in Japan: A nine‐year overview

**DOI:** 10.1002/pcn5.70368

**Published:** 2026-07-16

**Authors:** Hiroyuki Muraoka, Keisuke Mori, Takashi Tsuboi, Hitoshi Iida, Kentaro Fukumoto, Kazutaka Ohi, Kayo Ichihashi, Jun‐ichi Iga, Yuka Yasuda, Toshinori Nakamura, Eiichi Katsumoto, Tatsuya Nagasawa, Shusuke Numata, Shinichiro Ochi, Hirotaka Yamagata, Tomoyasu Wakuda, Masahiro Takeshima, Fumitoshi Kodaka, Yasushi Kawamata, Toru Horinouchi, Kazuhiko Yamamuro, Yusuke Arai, Satsuki Ito, Junya Matsumoto, Hisashi Yamada, Hikaru Hori, Ken Inada, Koichiro Watanabe, Norio Yasui‐Furukori, Ryota Hashimoto

**Affiliations:** ^1^ Department of Psychiatry Kitasato University, School of Medicine Kanagawa Japan; ^2^ Department of Pathology of Mental Diseases, National Institute of Mental Health National Center of Neurology and Psychiatry Tokyo Japan; ^3^ Department of Psychiatry The Jikei University School of Medicine Tokyo Japan; ^4^ Department of Neuropsychiatry Kyorin University School of Medicine Tokyo Japan; ^5^ Department of Psychiatry, Faculty of Medicine Fukuoka University Fukuoka Japan; ^6^ Department of Neuropsychiatry, School of Medicine Iwate Medical University Iwate Japan; ^7^ Department of Psychiatry Gifu University Graduate School of Medicine Gifu Japan; ^8^ Department of Neuropsychiatry University of Tokyo Hospital Tokyo Japan; ^9^ Department of Neuropsychiatry Ehime University Graduate School of Medicine Ehime Japan; ^10^ Life Grow Brilliant Mental Clinic Medical Corporation Foster Osaka Japan; ^11^ Department of Psychiatry Shinshu University School of Medicine Nagano Japan; ^12^ Katsumoto Mental Clinic Osaka Osaka Japan; ^13^ Department of Neuropsychiatry Kanazawa Medical University Ishikawa Japan; ^14^ Department of Psychiatry, Graduate School of Biomedical Science Tokushima University Tokushima Japan; ^15^ Department of Psychiatry Hamamatsu University School of Medicine Shizuoka Japan; ^16^ Department of Neuropsychiatry Akita University Graduate School of Medicine Akita Japan; ^17^ Department of Psychiatry Dokkyo Medical University, School of Medicine Tochigi Japan; ^18^ Department of Psychiatry and Neurology Hokkaido University Hospital Hokkaido Japan; ^19^ Center for Health Control Nara Medical University Nara Japan; ^20^ Department of Psychiatry Nara Medical University Nara Japan; ^21^ Department of Community Mental Health Shinshu University School of Medicine Nagano Japan; ^22^ Department of Neuropsychiatry Hyogo Medical University Hyogo Japan

**Keywords:** clinical practice guideline, dissemination, education, major depressive disorder, schizophrenia

## Abstract

**Aim:**

This study aimed to describe the nationwide reach, longitudinal growth, and geographic dissemination of the Effectiveness of Guideline for Dissemination and Education in Psychiatric Treatment (EGUIDE) project in Japan from 2016 to 2024.

**Methods:**

The EGUIDE project is a nationwide, multicenter implementation initiative designed to promote the dissemination of clinical practice guidelines, specifically the Guidelines for Pharmacological Treatment of Schizophrenia and the Treatment Guidelines II: Major Depressive Disorder. The project provides educational lectures primarily for psychiatrists and other mental health professionals. We descriptively summarized the cumulative numbers of lectures delivered, participants enrolled, and participating institutions from 2016 to 2024. In addition, we calculated the proportion of hospitals with psychiatric departments that participated in each prefecture.

**Results:**

Between 2016 and 2024, a total of 194 guideline lectures on schizophrenia and major depressive disorder were conducted. The cumulative number of participants reached 4785 across 763 institutions, of which 592 were hospitals with psychiatric departments, corresponding to approximately 20% of all such hospitals nationwide. Participating hospitals were distributed across most prefectures in Japan, indicating broad geographic coverage.

**Conclusion:**

Over the 9‐year period, the EGUIDE project continuously delivered guideline lectures and steadily expanded its participant and institutional base. The nationwide distribution of participating institutions suggests that the project has established a broad implementation platform for psychiatric guideline dissemination in Japan. Continued efforts may further strengthen this dissemination network and support future evaluations of its impact on psychiatric practice.

## INTRODUCTION

Clinical practice guidelines are developed based on scientific evidence and are intended to support shared decision‐making between patients and clinicians regarding treatment plans.^1^ Major clinical guidelines for psychiatric disorders recommend antipsychotic monotherapy for patients with schizophrenia and antidepressant monotherapy for patients with major depressive disorder (MDD).^2^, ^3^, ^4^, ^5^ However, these recommendations have not been widely adopted in routine clinical practice, and a gap between guideline‐recommended treatments and routine clinical practice (the Evidence–Practice Gap) has been noted.^6^ As a result, the frequent use of polypharmacy in routine clinical practice has become a significant concern.^7^, ^8^ To resolve this problem, the implementation of clinical practice guidelines into routine clinical practice is essential.

Educational interventions for clinicians (e.g., academic detailing) and reminders (such as audit and feedback) have long been recognized as effective strategies for implementing clinical practice guidelines. However, high‐quality evidence remains scarce, and the extent to which these established methodologies are truly effective is still unclear.^9^ The Effectiveness of Guideline for Dissemination and Education in Psychiatric Treatment (EGUIDE) project is a nationwide, multicenter initiative launched in Japan in 2016 as a strategy to support the implementation of psychiatric treatment guidelines in real‐world practice.^10^ The EGUIDE project organizes educational lectures on psychiatric treatment guidelines for clinicians, primarily psychiatrists, and collects patient treatment data from participating institutions as a platform for evaluating implementation and practice patterns over time. Previous studies have shown that psychiatrists who attend the guideline lectures report high satisfaction, improved understanding of the guidelines, and better adherence to selected guideline‐recommended practices. In addition, prior reports from participating institutions have suggested gradual increases in several guideline‐related quality indicators over time.^10^, ^11^, ^12^, ^13^, ^14^ Taken together, these prior findings provide the background for the ongoing national dissemination of the EGUIDE project. In this paper, we describe the nationwide reach, expansion, and geographic spread of the guideline lectures over the 9‐year period from 2016 to 2024.

## METHODS

This study was designed as a descriptive analysis of the nationwide dissemination of the EGUIDE project rather than an inferential evaluation of clinical outcomes or quality improvement.

The guideline lectures are intensive, 1‐day educational programs, with separate sessions offered for schizophrenia and MDD. The guidelines covered in these lectures include: the Guidelines for Pharmacological Treatment of Schizophrenia (2016–2021),^15^ the Guidelines for Pharmacological Treatment of Schizophrenia 2022 (2022–),^16^ Treatment Guidelines II: Major Depressive Disorder (2016–),^17^ Clinical Guidelines for Pregnant and Postpartum Women with or at Risk for Psychiatric Disorders (2021–),^18^ Prevention Guide for Obesity and Diabetes in patients with Schizophrenia (2021–),^19^ and Guidelines for Diagnosis and Treatment of Depression in Older Adults (2021–).^20^ Most of the lectures are conducted in person, although some are held online. The primary participants are psychiatrists, but other clinicians, including pharmacists, psychologists, and nurses, also attend. Details of the lectures have been reported in previous studies.^10^ The cumulative number of participants was calculated based on all attendees of the guideline lectures during the study period. Participating institutions were defined as institutions to which the attendees belonged. For the prefectural analysis, participating hospitals with psychiatric departments were identified for each prefecture, and the participation rate was calculated by dividing the number of participating hospitals by the total number of hospitals with psychiatric departments in the corresponding prefecture, based on the 2023 Survey of Medical Institutions.^21^ R version 4.4.0 was used to generate the map of Japan.

For patient treatment data, cumulative numbers were calculated annually for patients with schizophrenia and patients with MDD, respectively. Detailed information on the treatment data has been reported in previous studies.^10^ Participation in the guideline lectures was voluntary, and written informed consent was obtained. Collection of patient treatment data was conducted with an opt‐out approach. This study was approved by the Ethics Committee of the National Center of Neurology and Psychiatry. The protocol of the EGUIDE project has been registered in the University Hospital Medical Information Network (UMIN000022645). The study was conducted in accordance with the Declaration of Helsinki of the World Medical Association.

## RESULTS

**Figure 1:**
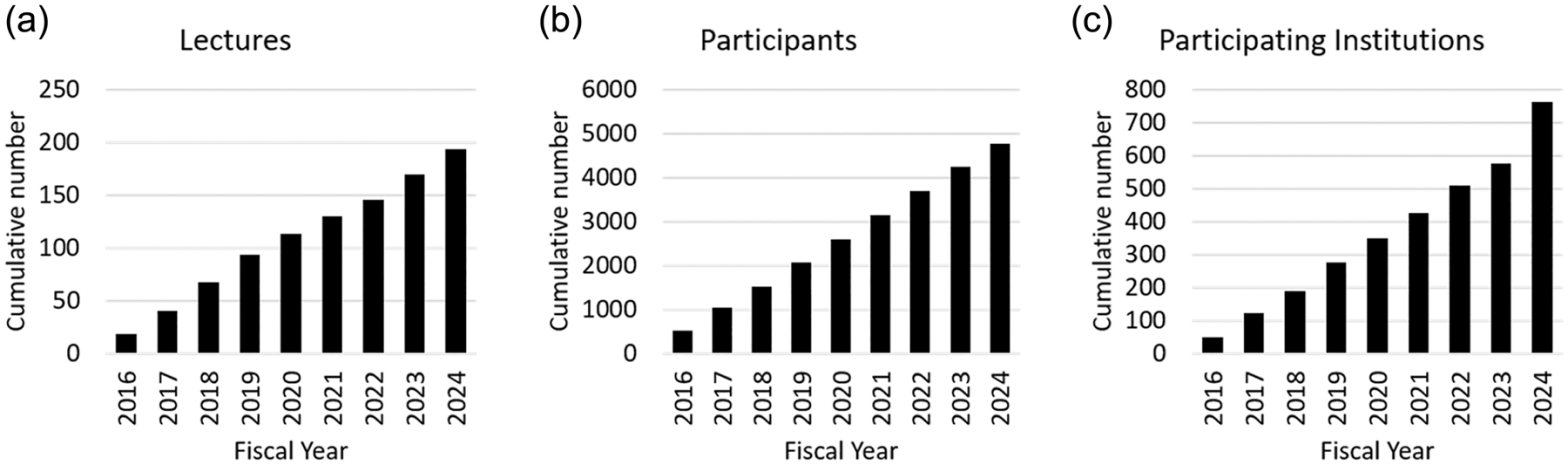
Cumulative totals of lectures, participants, and participating institutions from 2016 to 2024. (a) Cumulative total of lectures, (b) cumulative total of participants, and (c) cumulative total of participating institutions.

Between 2016 and 2024, a total of 194 guideline lectures on schizophrenia and MDD were conducted (Figure 1a). The number of participants increased by approximately 500 each year, reaching a cumulative total of 4785 participants (Figure 1b). Demographic information, including years of psychiatric experience, was available for a subset of 1840 participants. Among these participants, the median years of psychiatric experience was 1 year (interquartile range, 1–3 years; mean ± SD, 3.6 ± 5.6 years). The distribution was as follows: 1–5 years, 1520 participants (82.6%); 6–10 years, 152 (8.3%); 11–15 years, 61 (3.3%); 16–20 years, 50 (2.7%); 21–30 years, 44 (2.4%); and ≥31 years, 13 (0.7%).

The number of institutions to which participants belonged also increased along with the growing number of participants, reaching a cumulative total of 763 institutions (Figure 1c). Institutional classification was available for 758 of the 763 participating institutions. Based on available institutional names and information, these institutions were descriptively classified as follows: university hospitals, 89; general hospitals, 162; public psychiatric hospitals, 36; private psychiatric hospitals, 305; clinics, 146; and other institutions, 20. Five institutions could not be classified. Because some institutions may have overlapping characteristics, this classification reflects the main institutional characteristics available to us and does not necessarily indicate whether each institution had a psychiatric department.

**Figure 2:**
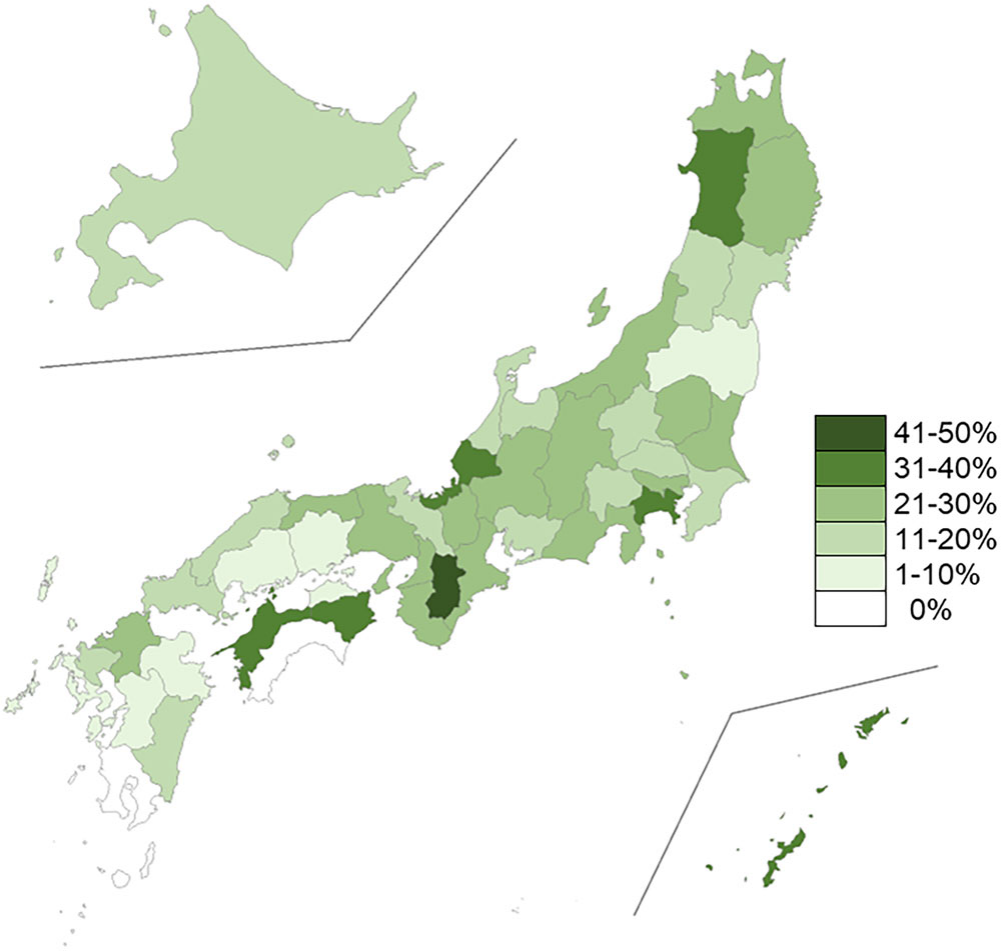
Distribution of participating hospitals with psychiatric departments by prefecture. The gradient bar indicates the participation rate, defined as the number of Effectiveness of Guideline for Dissemination and Education in Psychiatric Treatment (EGUIDE)‐participating hospitals with psychiatric departments divided by the total number of hospitals with psychiatric departments in each prefecture, based on the 2023 Survey of Medical Institutions.^21^

Separately, for the prefectural coverage analysis, 592 participating institutions were identified as hospitals with psychiatric departments; given that there were 2881 hospitals with psychiatric departments nationwide in 2023, this indicates that participants in the EGUIDE project were employed at approximately 20% of such hospitals in Japan. The prefectural distribution of participating hospitals with psychiatric departments is shown in Figure 2. Participating hospitals were identified in 45 of the 47 prefectures. The participation rate of hospitals with psychiatric departments varied across prefectures, ranging from 0.0% to 41.7% (Figure 2). Participation rates were relatively high in some prefectures and low in others, suggesting that there is still room for further expansion of the project.

**Figure 3:**
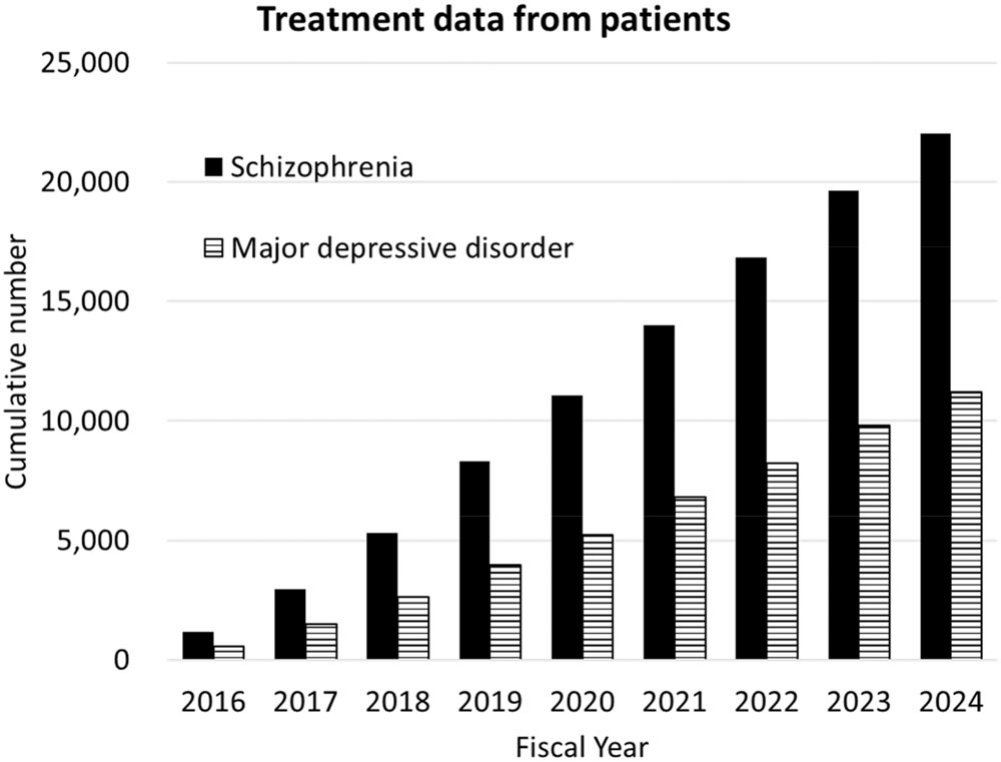
Cumulative numbers of patient treatment records for schizophrenia and major depressive disorder, 2016–2024. The black bars represent patients with schizophrenia, while the striped bars represent patients with major depressive disorder.

Patient treatment data were collected at admission and discharge. For patients with schizophrenia, approximately 2000–3000 cases were added each year, resulting in a cumulative total of 22,032 cases (Figure 3). For patients with MDD, approximately 1000–1500 cases were added annually, yielding a cumulative total of 11,207 cases (Figure 3).

## DISCUSSION

The guideline lectures were conducted continuously over a 9‐year period, with the numbers of both participants and participating institutions steadily increasing over time. As shown by the distribution of participating hospitals with psychiatric departments across Japan (Figure 2), the EGUIDE project has achieved broad nationwide reach and geographic dissemination. One factor that may have supported the nationwide dissemination of the guideline lectures was their continuous implementation. In general, once activities of this kind are interrupted, resumption can be difficult; therefore, sustained annual implementation may have contributed to the expansion of the program. According to the 2022 Overview of Statistics on Physicians, Dentists, and Pharmacists published by the Ministry of Health, Labour and Welfare, 16,817 psychiatrists work in medical institutions in Japan.^22^ Because most participants attended both the schizophrenia and MDD sessions, approximately 2400 psychiatrists are estimated to have participated in the EGUIDE project over the 9‐year period. This corresponds to about 14% of psychiatrists nationwide. If the current trend continues, approximately 1.5% of psychiatrists nationwide may newly participate each year, and in 10 years, roughly 30% may have attended the guideline lectures.

The EGUIDE project started in 2016 with 16 university hospitals. Initially, recruitment focused on university hospitals and then gradually expanded to national and public psychiatric hospitals as well as private psychiatric hospitals. However, many of these participating institutions are affiliated teaching hospitals that actively train early‐career psychiatrists. For broader dissemination of the guidelines, it will also be important to engage psychiatrists working in private psychiatric hospitals and clinics that are not affiliated with university hospitals. Since around 2021, the project has also introduced online workshops targeting psychiatrists working primarily in private clinics. By broadening the target audience, the number of new participants may increase, and the overall dissemination rate may eventually exceed the projected 30% estimated over the next 10 years. In addition, the influence of the guideline lectures may extend beyond individual participants and may produce a ripple effect within their affiliated institutions.^14^, ^23^ Therefore, the actual dissemination of the guidelines may be broader than that suggested by the number of participating psychiatrists alone. Nevertheless, nationwide dissemination remains incomplete, underscoring the need for continued project activities. To support long‐term sustainability, future efforts should consider strategies for integrating the guideline lectures into formal psychiatric training. The marked regional variation observed in participation rates may reflect differences in institutional networks, the presence of university‐affiliated hospitals, and access to training opportunities across prefectures. In particular, prefectures in which university hospitals joined the project during its early phase may have been more likely to develop broader local participation. At the same time, lower participation rates in some prefectures suggest that further efforts are needed to improve accessibility and outreach. Although the present study did not directly examine the determinants of regional variation, the continued expansion of online workshops and other outreach strategies may help reduce these geographic disparities in future implementation.

A notable secondary outcome of the nationwide expansion of the EGUIDE project has been the accumulation of treatment data from approximately 33,000 patients, enabling a comprehensive assessment of clinical practice. For example, the project has generated findings on the use of antipsychotics in combination with other psychotropic medications,^24^ anxiolytics and hypnotics,^25^, ^26^ anticholinergic agents,^27^, ^28^ long‐acting injectable antipsychotics,^29^ and as‐needed psychotropic medications,^30^, ^31^ thereby providing insight into prescribing patterns, including polypharmacy, in Japanese psychiatric care. To our knowledge, such multiregional, multi‐institutional surveys using real‐world data on schizophrenia and MDD remain uncommon in Japan. This study has several limitations. First, this was a descriptive report of nationwide dissemination and did not include inferential statistical testing. Second, the present analysis did not directly evaluate changes in clinical outcomes or quality indicators over time. Third, participating institutions were not randomly selected and may have included centers with a greater interest in guideline‐based care, which may limit generalizability. Fourth, we did not examine inter‐institutional variability, disorder‐specific differences, or the effects of participation intensity, institutional characteristics, or lecture format. In addition, although prefectural variation in participation rates was observed, the present analysis did not investigate the factors underlying these differences. Finally, the number of participants and participating institutions does not necessarily reflect the depth of implementation in routine clinical practice. Despite these limitations, the present findings provide a useful overview of the national reach and sustained expansion of the EGUIDE project over a 9‐year period.

In summary, the guideline implementation strategy of the EGUIDE project has expanded nationwide and has established a broad platform for the dissemination of psychiatric treatment guidelines in Japan. Although the present study did not directly evaluate changes in clinical outcomes or quality indicators, the project may serve as a useful model for the implementation of guidelines for other psychiatric disorders, such as bipolar disorder and anxiety disorders. The experience gained through this project may also inform future guideline dissemination efforts in other clinical fields and settings. Future studies should examine the impact of the EGUIDE project on clinical outcomes and identify the factors that facilitate or hinder guideline implementation at the institutional and regional levels.

## AUTHOR CONTRIBUTIONS

Hiroyuki Muraoka was involved in data collection and analysis and manuscript preparation. Keisuke Mori was involved in analysis and manuscript preparation. Takashi Tsuboi, Hitoshi Iida, Kentaro Fukumoto, Kazutaka Ohi, Kayo Ichihashi, Jun‐ichi Iga, Yuka Yasuda, Toshinori Nakamura, Eiichi Katsumoto, Tatsuya Nagasawa, Shusuke Numata, Shinichiro Ochi, Hirotaka Yamagata, Tomoyasu Wakuda, Masahiro Takeshima, Fumitoshi Kodak, Yasushi Kawamata, Toru Horinouchi, Kazuhiko Yamamuro, Yusuke Arai, Satsuki Ito, Junya Matsumoto, Hisashi Yamada, Hikaru Hori, and Norio Yasui‐Furukori contributed to the interpretation of the data and data collection. Koichiro Watanabe and Ken Inada were involved in the study design and contributed to the interpretation of the data. Ryota Hashimoto supervised the entire project and was involved in data collection, the study design, analysis, and interpretation of the data. All authors contributed to and approved the final article.

## CONFLICT OF INTEREST STATEMENT

Norio Yasui‐Furukori is an Editor‐in‐Chief of *Psychiatry and Clinical Neurosciences Reports*, and Yasushi Kawamata is a Deputy Managing Editor of *Psychiatry and Clinical Neurosciences Reports*. They were not involved in the editorial evaluation or decision‐making process for this manuscript. The remaining authors declare no conflicts of interest.

## ETHICS APPROVAL STATEMENT

The study was conducted with the approval of the National Center of Neurology and Psychiatry Ethics Committee. The study was conducted in accordance with the Declaration of Helsinki of the World Medical Association.

## PATIENT CONSENT STATEMENT

Written informed consent was obtained from each participating psychiatrist. Patient treatment data were collected at each participating facility using an opt‐out approach.

## CLINICAL TRIAL REGISTRATION

The protocol for the EGUIDE project is registered with the University Hospital Medical Information Network Registry (UMIN000022645).

## Data Availability

The data are not publicly available due to privacy and ethical restrictions (i.e., we did not obtain informed consent on the public availability of raw data).
